# Molecular characterization of invasive *Neisseria meningitidis* isolates collected in Lithuania (2009-2019) and estimation of serogroup B meningococcal vaccine 4CMenB and MenB-Fhbp coverage

**DOI:** 10.3389/fcimb.2023.1136211

**Published:** 2023-02-15

**Authors:** Emilija Sereikaitė, Rūta Plepytė, Aurelija Petrutienė, Dovilė Stravinskienė, Indrė Kučinskaitė-Kodzė, Vilmantas Gėgžna, Inga Ivaškevičienė, Aurelija Žvirblienė, Milda Plečkaitytė

**Affiliations:** ^1^ Institute of Biotechnology, Life Sciences Center, Vilnius University, Vilnius, Lithuania; ^2^ Department of Bacteriology, National Public Health Surveillance Laboratory, Vilnius, Lithuania; ^3^ Institute of Biosciences, Life Sciences Center, Vilnius University, Vilnius, Lithuania; ^4^ Clinic of Children’s Diseases, Institute of Clinical Medicine, Faculty of Medicine, Vilnius University, Vilnius, Lithuania; ^5^ Pediatric Center, Vilnius University Hospital Santaros Klinikos, Vilnius, Lithuania

**Keywords:** *Neisseria meningitidis*, serogroup B, invasive meningococcal disease, multilocus sequence typing, gMATS, MenDeVAR Index, vaccine coverage

## Abstract

*Neisseria meningitidis* causes invasive meningococcal disease (IMD), which is associated with significant mortality and long-term consequences, especially among young children. The incidence of IMD in Lithuania was among the highest in European Union/European Economic Area countries during the past two decades; however, the characterization of meningococcal isolates by molecular typing methods has not yet been performed. In this study, we characterized invasive meningococcal isolates (n=294) recovered in Lithuania from 2009 to 2019 by multilocus sequence typing (MLST) and typing of antigens FetA and PorA. The more recent (2017-2019) serogroup B isolates (n=60) were genotyped by analyzing vaccine-related antigens to evaluate their coverage by four-component (4CMenB) and two-component (MenB-Fhbp) vaccines using the genetic Meningococcal Antigen Typing System (gMATS) and Meningococcal Deduced Vaccine Antigen Reactivity (MenDeVAR) Index methods, respectively. The vast majority (90.5%) of isolates belonged to serogroup B. MLST revealed a predominance of clonal complex 32 (74.02%). Serogroup B strain P1.19,15: F4-28: ST-34 (cc32) accounted for 64.1% of IMD isolates. The overall level of strain coverage by the 4MenB vaccine was 94.8% (CI 85.9-98.2%). Most serogroup B isolates (87.9%) were covered by a single vaccine antigen, most commonly Fhbp peptide variant 1 (84.5% of isolates). The Fhbp peptides included in the MenB-Fhbp vaccine were not detected among the analyzed invasive isolates; however, the identified predominant variant 1 was considered cross-reactive. In total, 88.1% (CI 77.5-94.1) of isolates were predicted to be covered by the MenB-Fhbp vaccine. In conclusion, both serogroup B vaccines demonstrate potential to protect against IMD in Lithuania.

## 1 Introduction

The Gram-negative bacterium *Neisseria meningitidis* is a commensal of the nasopharynx in up to 35% of healthy individuals at any given time (Coureuil et al., 2012; Parikh et al., 2020). It is not fully understood why meningococci in some colonized individuals may pass the mucosal barrier and enter the bloodstream, resulting in septicemia (Coureuil et al., 2012; Schoen et al., 2014). Their further proliferation in the bloodstream may progress to cardiovascular failure and dissemination to other organs*. N. meningitidis* crosses the blood-brain barrier in about 60% of bacteremia cases causing meningitis (Miller et al., 2013; Nadel and Ninis, 2018). Thus, rapidly evolving invasive meningococcal disease (IMD) is a life-threatening condition that causes significant morbidity and mortality, especially in children and young adults (Nadel and Ninis, 2018).

*N. meningitidis* may be either unencapsulated or encapsulated, the latter which is divided into 12 serogroups based on different capsular polysaccharide structures (Centers for Disease Control and Prevention, 2016; Whittaker et al., 2017). Only six meningococcal serogroups (A, B, C, W, Y, and X) cause the majority of IMD cases worldwide; however, serogroup distribution and the incidence of IMD vary by continent and region (Bai et al., 2019; Parish et al., 2020). To perform active surveillance of IMD outbreaks and sporadic cases, molecular techniques that precisely characterize invasive and carried meningococci have been developed. Multilocus sequence typing (MLST) allows determination of the sequence type (ST) and clonal complex (cc) of meningococcal isolates (Maiden et al., 1998; Jolley et al., 2007). Typing of several surface proteins (PorA, PorB, and FetA) is also recommended (Jolley et al., 2007). Recently, the Genetic Meningococcal Typing System (gMATS) (Muzzi et al., 2019) and Meningococcal Deduced Vaccine Antigen Reactivity (MenDeVAR) Index methods (Rodrigues et al., 2020) based on sequence analysis of vaccine antigen variants in meningococcal isolates open the possibility of predicting serogroup B (MenB) strain coverage by available meningococcal B vaccines.

In Lithuania, which has 2.8 million inhabitants, the incidence of IMD cases was among the highest in European Union/European Economic Area countries during 2009-2019, reaching 3 and 2.9 cases/10^5^ in 2013 and 2017, respectively (National Public Health Center under the Ministry of Health, 2021). Serogroup B is responsible for most IMD cases in Lithuania (National Public Health Center under the Ministry of Health, 2021), but invasive *N. meningitidis* isolates have not been characterized by molecular typing methods. In 2018, the only four-component meningococcal B vaccine (4CMenB) was introduced to Lithuania’s National Immunization program and administered at 3, 5, and 12-15 months of age. The two-component meningococcal B vaccine (MenB-Fhbp) is registered for the vaccination of individuals 10 years and older, but the cost of the vaccine is not reimbursed. Studies on the potential coverage of MenB vaccines in Lithuania would contribute to better monitoring of vaccination impact and planning of future prophylactic strategies.

The aim of this study was to characterize invasive *N. meningitidis* isolates collected in Lithuania between 2009-2019 by molecular typing and to evaluate the potential 4CMenB and MenB-Fhbp vaccine coverage of isolates from 2017-2019 IMD cases.

## 2 Materials and methods

### 2.1 Meningococcal isolates

All cases of IMD require mandatory reporting in Lithuania. Since 2009, invasive *N. meningitidis* isolates are requested to be sent to the National Public Health Surveillance Laboratory (NPHSL) (Legal acts of the Republic of Lithuania, Ministry of Health, 2009). The NPHSL received 316 invasive meningococcal isolates between 1 July 2009 and 31 Dec 2019. All meningococci were recovered from the blood or cerebrospinal fluid (CSF) of individuals with IMD. Nine isolates could not be cultured and were excluded from this study. If more than one isolate was received from the same individual, only one isolate was included. A total of 294 N*. meningitidis* isolates were analyzed. Information on the gender and age of the individual and the region of isolation was obtained from the NPHSL. The study was approved by the Vilnius Regional Biomedical Research Ethics Committee (http://www.eurecnet.org/information/lithuania.html), approval no. 2021/9-1369-846, issued 21-09-2021.

### 2.2 Molecular characterization of *N. meningitidis* isolates

Meningococcal isolates were stored frozen (-80°C) at the NPHSL. For this study, isolates were cultured on Chocolate agar (Oxoid, UK) for 12-14 h at 36°C and 5% CO_2_. DNA from cultured isolates was extracted using a GeneJET genomic DNA purification kit (Thermo Fisher Scientific, Vilnius, Lithuania) according to the manufacturer’s instructions. About 20 ng of purified chromosomal DNA was used as a template for amplification of all genes. Meningococcal isolates were serogrouped by slide agglutination by the isolate providers. Serogroup verification was performed by singleplex polymerase chain reaction (PCR)-based assay (Fraisier et al., 2009; Zhu et al., 2012). PCR was performed with Phire Green Hot Start II PCR Master Mix (Thermo Fisher Scientific, Vilnius, Lithuania) in 20-µL reaction volume comprising 0.4 µM of each primer (Metabion International AG, Germany). Amplification reactions included initial denaturation at 98°C for 30 s, 30 amplification cycles consisting of denaturation for 5 s at 98°C, annealing for 5 s at 60°C, and extension for 10 s at 72°C. These were followed by a final extension step for 1 min at 72°C. No template controls were included in the PCR assays. Amplification products were separated on 2.5% agarose gel.

MLST, PorA, and FetA typing for isolates received in 2009-2017 were performed according to the procedure described on the PubMLST *Neisseria* website (http://pubmlst.org/neisseria/; Jolley et al., 2018). The amplification enzymes used were Phire Hot Start II DNA polymerase included in the master mix (Thermo Fisher Scientific) in the case of *porA* and *fetA*, and DreamTaq Hot Start DNA polymerase included in the master mix (Thermo Fisher Scientific) for each of the MLST housekeeping genes. The reactions were performed in a 25-µL final volume. PCR conditions for amplification of *porA* and *fetA* were, as follows: initial denaturation at 98°C for 30 s, 30 amplification cycles consisting of denaturation for 5 s at 98°C, annealing for 5 s at 60°C, and extension for 15 s at 72°C. A final extension step lasted for 1 min at 72°C. Amplification reaction mixture with DreamTaq Hot Start DNA polymerase was subjected to initial denaturation at 95°C for 3 min, 35 cycles of denaturation at 95°C for 30 s, primer annealing at 57°C (for *abcZ, adk, aroE, fumC*, and *pdhC*) or 67°C (for *pgm*) for 30 s, extension at 72°C for 40 s, followed by a final extension step at 72°C for 7 min. Amplification products were analyzed on 1.5% agarose gel.

PCR products were purified using a DNA Clean & Concentrator kit (Zymo Research, Irvin, CA, US) and subjected to Sanger sequencing (Base Clear B.V., Leiden, The Netherlands). Allele assignment and MLST ST and cc were obtained from the PubMLST *Neisseria* database. For isolates received in 2017, amplification of vaccine antigens *nhba, fhbp*, and *nadA* was performed as previously described (Bambini et al., 2009; Lucidarme et al., 2010). PCR reaction mixture with Phire Hot Start II DNA polymerase included in the master mix was subjected to initial denaturation at 98°C for 30 s, 30 amplification cycles consisting of denaturation for 5 s at 98°C, annealing for 5 s at 60°C (for *nhba* and *nadA*) or 63°C (for *fhbp*), extension for 15 s at 72°C, followed by a final extension step at 72°C for 1 min.

Internally directed primers nadaintF and nadaintR for detecting *nadA* were used as described by Lucidarme et al. (2009). Briefly, PCR was performed with Phire Hot Start II DNA polymerase included in the master mix using reaction conditions described for amplification of *porA* and *fetA*.

For isolates not subjected to whole genome sequencing (WGS), the presence/absence of *nadA* in the meningococcal genome was confirmed using internally directed primers in case the application of primers targeting the flanking regions did not yield any PCR product (Lucidarme et al., 2009). The PubMLST *Neisseria* database was used to assign allelic and peptide variants of vaccine antigens.

Isolates received in 2018-2019 (n=25) underwent WGS with support from the European Center for Disease Prevention and Control. WGS was conducted by Eurofins Genomics Europe Sequencing Gmbh (Konstanz, Germany) using the Illumina NovaSeq platform. Assembly was performed using SPAdes 3.11 (Bankevich et al., 2012) with careful mode enabled. Genome contigs were submitted to the PubMLST *Neisseria* database (see Data Availability Statement). The allelic profile of seven MLST genes and variable regions of *porA* and *fetA* were determined by automatic scanning of genome contigs (Jolley et al., 2018). Allelic and peptide variants of vaccine antigens (*fhbp*, *nhba*, and *nadA*) were also defined.

### 2.3 gMATS and the MenDeVAR Index

The gMATS method was used to predict strain coverage by the 4CMenB vaccine as described by Muzzi et al. (2019). Genes of vaccine antigens were obtained either by PCR and sequencing (isolates recovered in 2017) or WGS (isolates recovered in 2018-2019). Alleles and corresponding peptide identifiers were assigned by the PubMLST *Neisseria* database. Estimation of antigen-specific predicted coverage by 4CMenB was performed based on criteria described by Muzzi et al. (2019). NadA was not considered as contributing to coverage in gMATS (Muzzi et al., 2019).

The potential strain coverage by the MenB-Fhbp vaccine was assessed using the MenDeVAR Index method according to criteria outlined in Rodrigues et al. (2020).

### 2.4 Statistical analysis

Data were analyzed using the R statistical computer program, version 4.2.2 (R Core Team, 2017), and Microsoft Excel. Ninety-five percent confidence intervals (CIs) of proportions were calculated using Wilson’s method (Agresti and Coull, 1998) implemented in the R package DescTools, version 0.99.47, and then converted to percentages.

## 3 Results

### 3.1 Epidemiological background

During the study period 2009-2019, 745 cases of sporadic IMD were reported in Lithuania, corresponding to an overall IMD incidence of 2.24 cases/10^5^ inhabitants (National Public Health Center under the Ministry of Health, 2021). The highest incidence was observed in 2013, with 3 cases/10^5^ inhabitants (National Public Health Center under the Ministry of Health, 2021) (**Table 1**). The largest number of IMD cases was observed in <5-year-olds (47.5%), whereas ≥30-year-olds accounted for 18.1% of cases. The worst disease-affected region was the Vilnius region, accounting for the majority of disease cases across all age groups (National Public Health Center under the Ministry of Health, 2021).

**Table 1:** Distribution of IMD cases by year and age.

Year	2009	2010	2011	2012	2013	2014	2015	2016	2017	2018	2019
Cases	65	50	80	83	89	71	74	75	81	40	37
Cases/10^5^ population	1.9	1.5	2.5	2.8	3.0	2.4	2.5	2.6	2.9	1.4	1.3
Number of isolates stored at the NPHSL	8 (12.3%*)	12 (24%)	26 (32.5%)	31 (35.8%)	38 (42.7%)	33 (46.5%)	39 (52.7%)	40 (53.3%)	42 (51.8%)	10 (25%)	15 (40.5%)
Age group											
<1 y	15 (2**)	13 (1)	7 (3)	16 (1)	25 (3)	14 (2)	9 (0)	14 (4)	12 (3)	3 (1)	3 (1)
1-4 y	24 (0)	22 (3)	20 (1)	23 (2)	20 (3)	20 (2)	26 (3)	21 (1)	22 (0)	15 (3)	10 (1)
5-14 y	10 (0)	5 (0)	15 (0)	14 (0)	11 (0)	13 (0)	20 (3)	8 (1)	15 (1)	9 (0)	4 (0)
15-29 y	9 (0)	4 (0)	26 (2)	19 (1)	11 (2)	11 (0)	10 (0)	13 (1)	15 (1)	7 (0)	7 (1)
≥30 y	7 (3)	6 (3)	12 (1)	11 (5)	22 (3)	13 (1)	9 (1)	19 (1)	17 (5)	6 (1)	13 (3)
Mortality/10^5^ population	0.2	0.2	0.2	0.3	0.4	0.2	0.2	0.3	0.4	0.2	0.2

*Proportion (%) of isolates from annually reported IMD cases stored at the NPHSL and analyzed in this study.

**Value inside parenthesis indicates number of deaths.

The proportion of cases diagnosed by PCR without culture is unknown. The clinical guidelines (Diagnostic and Treatment Protocols, Ministry of the Republic of Lithuania, 2015) recommend obtaining a sample of blood and/or CSF for culturing. The 294 isolates analyzed in this study were from culture-proven IMDs, which accounted for 39.4% of all reported IMD cases during the study period (**Table 1**). The highest proportion of isolates (51-53%) from annually reported IMD cases was in 2015-2017, while the lowest was in 2009-2010, which could be related to adapting to the recently introduced requirement to send invasive isolates to NPHSL (Legal acts of the Republic of Lithuania, Ministry of Health, 2009).

The vast majority of cases (90.5%, n=266) were caused by *N. meningitidis* serogroup B (**Table 2**). Serogroups C, Y, and W accounted for 7.5% (n=22), 1% (n=3), and 0.68% (n=2) of cases, respectively. Serogroups B and C were detected in all age groups, whereas MenY and MenW caused disease mostly in adults (National Public Health Center under the Ministry of Health, 2021) (**Table 3**).

**Table 2:** Serogroup distribution of *N. meningitidis* isolates by year.

	Serogroup (No. of isolates)				
Year (No. of isolates)	B	C	W	Y	NG
2009 (n=8)	8	−	−	−	−
2010 (n=12)	12	−	−	−	−
2011 (n=26)	25	1	−	−	−
2012 (n=31)	28	3	−	−	−
2013 (n=38)	35	3	−	−	−
2014 (n=33)	29	4	−	−	−
2015 (n=39)	36	3	−	−	−
2016 (n=40)	33	4	2	−	1
2017 (n=42)	37	3	−	2	−
2018 (n=10)	10	−	−	−	−
2019 (n=15)	13	1	−	1	−

NG, not groupable.

**Table 3:** Serogroup distribution of *N. meningitidis* isolates by age group.

	Serogroup (No. of isolates)				
Age group (No. of isolates)	B	C	W	Y	NG
<1 (n=33)	30	3	−	−	−
1-4 (n=61)	54	7	−	−	−
5-14 (n=28)	27	1	−	−	−
15-24 (n=63)	58	2	−	2	1
25-44 (n=41)	36	4	1	−	−
45-64 (n=35)	30	4	1	−	−
65+ (n=25)	24	−	−	1	−
UN (n=8)	7	1	−	−	−

UN, age unknown; NG, not groupable.

### 3.2 MLST analysis and distribution of clonal complexes

All 294 isolates were subjected to MLST analysis. The MLST profile was incomplete for 13 isolates (4.4%) because the PCR product of the gene(s), mainly *fumC*/*pdhC*, was not obtained, whereas the subtypes of finetyping antigens PorA and FetA were determined. The 281 isolates with complete MLST profiles were grouped into 73 STs, of which 63 (86.3%) were detected only once. The most common ST was ST-34 (cc32), accounting for 64.8% (n=182, 59.0-70.1%) of all isolates. Thirty-four isolates (12.1%) belonged to 31 STs that were not assigned to any known cc (ccUA). Among 45 designated new STs, 20 STs were grouped into five ccs, of which the most frequent was cc18, whereas 25 STs were not associated with any cc.

**Figure 1:**
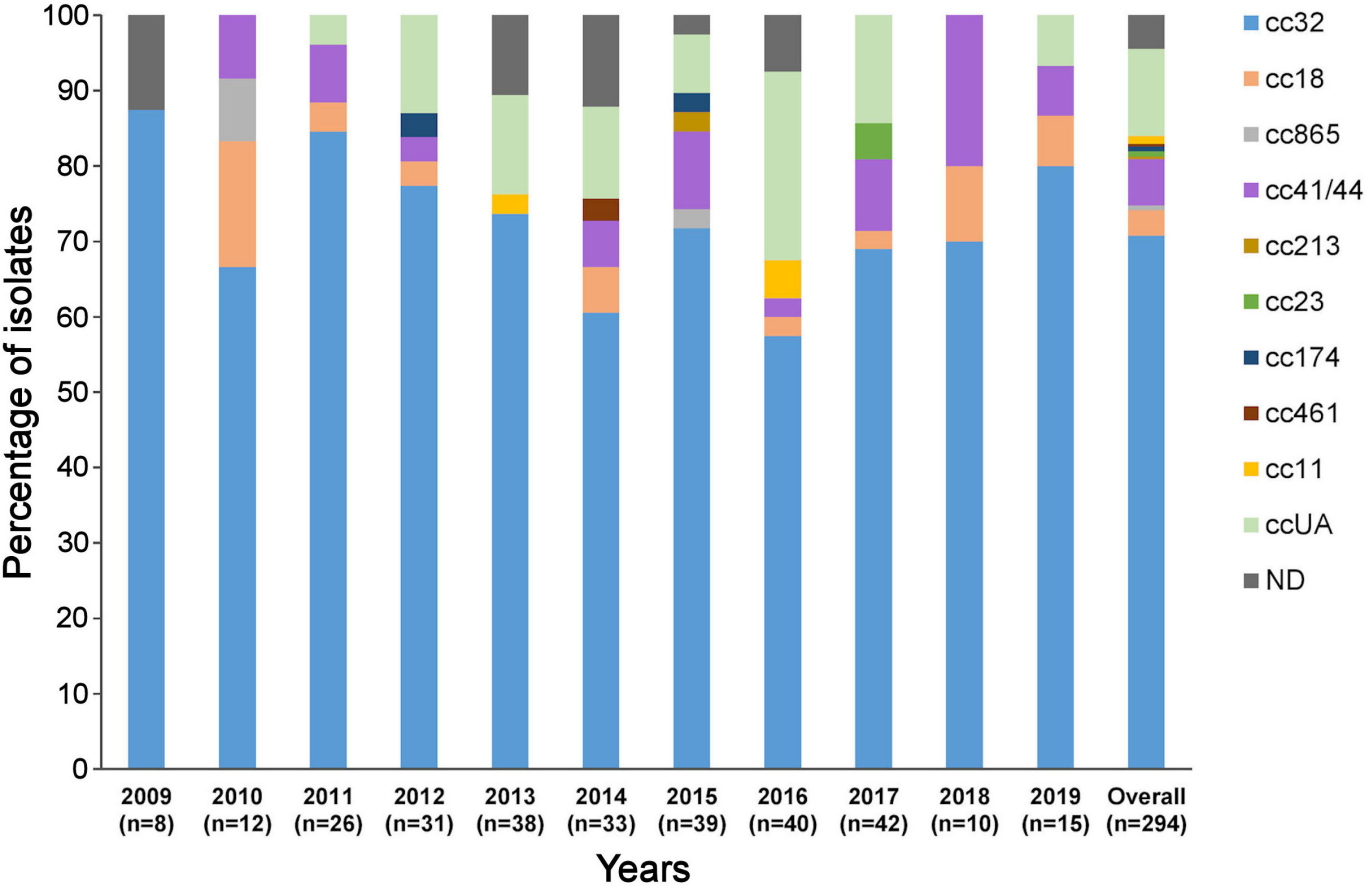
Distribution of clonal complexes in the panel of 294 isolates by year. ccUA, sequence type not assigned to any cc; ND, MLST profile not available; cc, clonal complex; n, number of isolates.

The isolates were assigned to nine ccs, the most frequent of which were cc32 (74.02%, n=208), cc41/44 (6.4%, n=18), and cc18 (3.6%, n=10) (**Figure 1**). The most common combination of PorA VR1/VR2 was P1.19,15, which belonged to cc32 isolates. Antigen finetyping showed that the serogroup B meningococcal strain P1.19,15: F4-28: ST-34 (cc32) was the most prevalent (64.1%, n=180) among IMD isolates.

**Figure 2:**
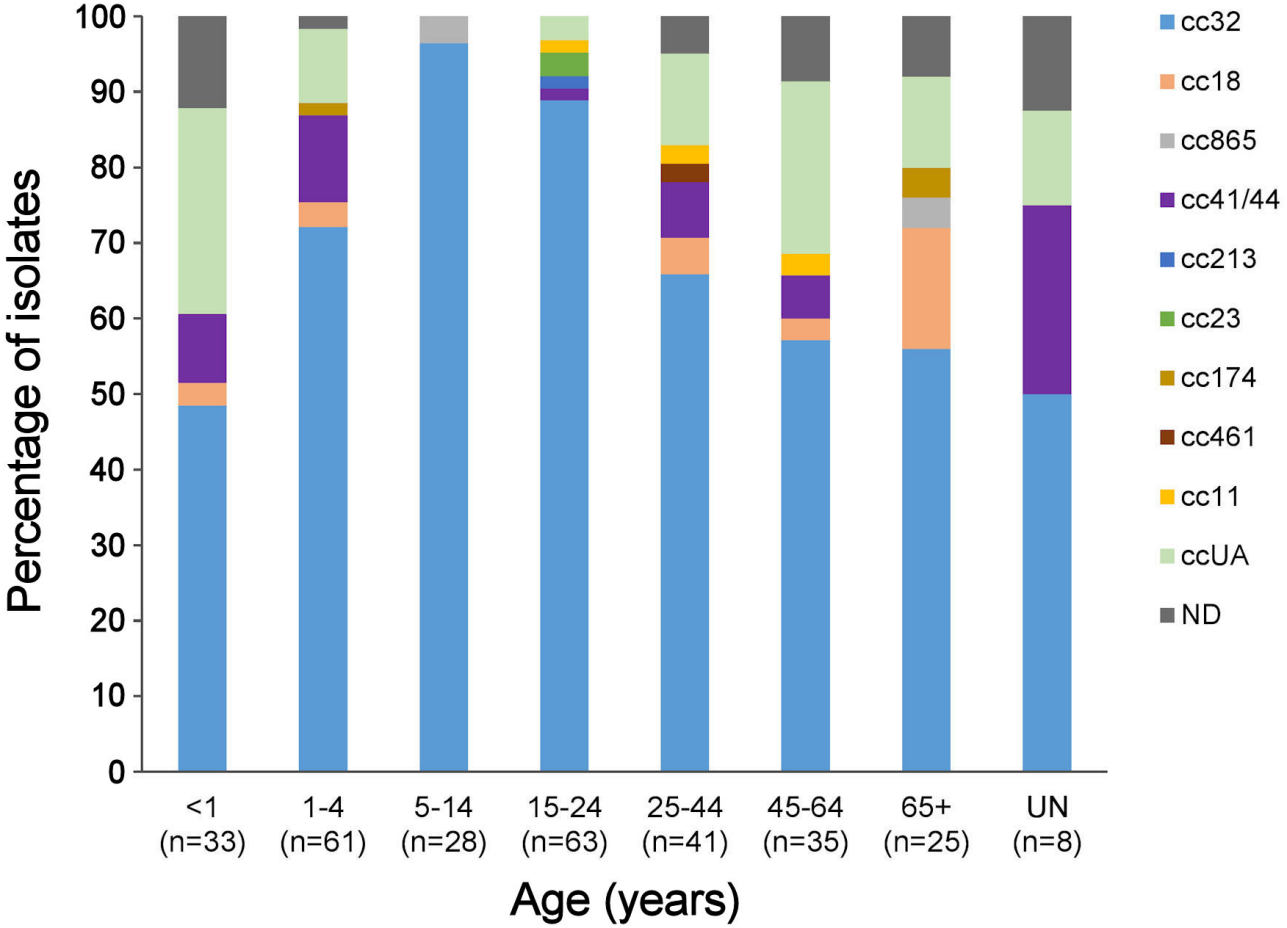
Distribution of clonal complexes according to age. ND, no age information of IMD cases (n=8); ccUA, sequence type not assigned to any cc; cc, clonal complex; n, number of isolates.

Generally, the meningococcal isolates in most age groups belonged to three different ccs, although cc32 was dominant (**Figure 2**). Among 5-14- and 15-24-year-olds, cc32 comprised 96.4% and 88.9% of isolates, respectively. Approximately 50% of isolates among children <1 year of age were attributed to cc32; however, 27.3% (CI 15.1-44.2%) of isolates were not assigned to any known cc. The proportion of ccUA was lower (9.8%) among 1-4-year-olds.

### 3.3 Molecular typing of vaccine antigens

The subtyping results of *fhbp, nhba*, and *nadA* genes performed for 60 MenB isolates of the IMD cases reported in 2017-2019 are shown in **Supplementary Table 1**.

For one isolate (ST-15979, cc18), the Fhbp peptide variant was not determined due to difficulties in obtaining the PCR product. Eleven *fhbp* alleles were found to encode 11 different peptide variants. Fhbp variant 1 (subfamily B), which is included in the 4CMenB vaccine, was found in 47 of 59 isolates (79.7%). The *nhba* gene could not be amplified for one isolate (ST-16207, ccUA). Four new *nhba* alleles were identified (1920, 1921, 1922, and 1930), three of which coded for the new Nhba peptide variants (1708, 1709, and 1715). The Nhba peptide variant 2 included in the 4CMenB vaccine was present in one isolate (ST-16176, ccUA), whereas the most prevalent peptide was variant 187 (47 of 59 isolates).

For one isolate (ST-16207, ccUA), the PCR product for PorA and Nhba was not obtained. The 4CMenB vaccine VR2:4 variant was found in two isolates attributed to cc41/44. For 29 isolates received in 2017, the PCR product of *nadA* was not obtained with previously described primers (Bambini et al., 2009; Lucidarme et al., 2010); therefore, the internally directed primers nadaintF and nadaintR (Lucidarme et al., 2009) were used to confirm the presence/absence of *nadA* elsewhere in the genome (**Supplementary Table 1**). The obtained PCR product of ~1,000 bp of *nadA*-positive isolates was verified by sequencing; however, the peptide variant was not determined. The NadA coding gene was detected in 48 of 60 isolates (80.0%), of which the majority were assigned to ST-34 (cc32). For 2018-2019 isolates for which WGS was performed, all *nadA*-positive isolates carried peptide variant 1 (**Supplementary Table 1**).

### 3.4 gMATS-based predicted coverage of MenB isolates by the 4CMenB vaccine

Vaccine coverage using gMATS (Muzzi et al., 2019) was estimated for 58 MenB isolates (2017-2019), as PCR of vaccine antigens was not obtained for two isolates (id 13530 and id 26292, **Supplementary Table 1**). gMATS predicted 53 (91.4%, CI 81.4-96.3%) covered isolates, 1 (1.7%) non-covered isolate, and 4 (6.9%) unpredictable isolates. Considering one-half of the unpredictable isolates as potentially covered, an overall level of coverage was 94.8% (55 of 58, CI 85.9-98.2%).

The predicted coverage of strains assigned to cc32 was 97.9% (47 of 48, CI 89.1-99.6%), and the antigen contributing to coverage was Fhbp peptide variant 1 (**Table 4**). Among the ‘unpredictable’ strains category, two ccUA isolates possessed alleles for Nhba peptide 42 and 53 and an allele for Fhbp peptide 13, one cc41/44 isolate possessed an allele for Nhbp peptide 1709, and one cc32 isolate possessed an allele for Fhbp peptide 180 (**Table 4**). Most MenB isolates (87.9%) were covered by a single antigen, namely Fhbp (84.5%) and Nhba (3.4%, peptides 2 and 20). One isolate was predicted to be covered by a combination of two antigens (Fhbp+PorA_VR2) and one isolate by three antigens (Fhbp+Nhba+PorA_VR2) (**Table 4**).

**Table 4:** The coverage of MenB isolates predicted by gMATS.

Clonal complex	Sequence type	Fhbp peptide variant	Nhbp peptide variant	PorA_VR variant	No. of isolates	Coverage by individual antigen and antigen combinations
cc32	ST-34	1	187	15	36	Fhbp coverage
cc32	ST-34	180	187	15	1	Unpredictable by Fhbp
cc32	ST-9775	1	187	15	4	Fhbp coverage
cc32	ST-9779	1	187	15	5	Fhbp coverage
cc32	ST-16171	1	187	15	1	Fhbp coverage
cc32	ST-16792	1	187	15	1	Fhbp coverage
cc41/44	ST-7670	14	1715	4	1	PorA_VR2+Fhbp coverage
cc41/44	ST-15968	4	2	4	1	PorA_VR2+Fhbp+Nhbp coverage
cc41/44	ST-15980	207	1709	28	1	Unpredictable by Nhbp and Fhbp
cc18	ST-4769	37	6	9	1	Fhbp covered
cc18	ST-9774	37	6	4-1	1	Fhbp covered
ccUA	ST-5477	13	53	28-7	1	Unpredictable by Nhbp and Fhbp
ccUA	ST-10261	647	42	28	1	Unpredictable by Nhbp
ccUA	ST-15982	119	6	10-4	1	Not covered
ccUA	ST-15983	321	20	1	1	Nhba covered
ccUA	ST-16176	207	2	28-5	1	Nhbp covered

### 3.5 MenDeVAR Index-based predicted coverage of MenB isolates by the MenB-Fhbp vaccine

A new tool, MenDeVAR Index (Rodrigues et al., 2020), was used to predict coverage of 59 MenB isolates by the MenB-Fhbp vaccine, as *fhbp* PCR product was not obtained for one isolate (ST-15979, cc18). All 59 isolates contained the *fhbp* gene, among which two isolates possessed an A/B hybrid variant (allele 231, peptide 207). The Fhbp peptides 45 and 55 included in the MenB-Fhbp vaccine were not detected among Lithuanian (2017-2019) isolates. The most frequent peptide 1 associated with cc32 was considered cross-reactive (‘amber’ MenDeVAR Index) (**Table 5**). There were insufficient data (‘grey’ Index) for the assessment of seven isolates associated with ccUA, cc18, and cc41/44. In total, 52 isolates from the analyzed 59 isolates (88.1%, CI 77.5-94.1) were considered potentially covered by the MenB-Fhbp vaccine (**Table 5**).

**Table 5:** Predicted coverage of MenB isolates by the MenB-Fhbp vaccine according to the MenDeVAR Index method.

MenDeVAR Index*	No. of isolates	% of isolates covered
Exact match (isolate contains exact vaccine antigen variants)	0	0
Cross-reactive (isolate contains cross-reactive vaccine antigen variants)	52	88.1
None (isolate contains neither exact vaccine antigen variants nor cross-reactive variants)	0	0
Insufficient data (insufficient data to make an assessment of the isolate’s vaccine antigen variants)	7	11.9

*Data interpretation using the traffic light colors was applied as described in the Materials and Methods section and **Table 2** by Rodrigues et al. (2020).

### 3.6 Comparison of predicted coverage of MenB isolates by the 4CMenB and MenB-Fhbp vaccines

Estimation of predicted 4CMenB and MenB-Fhbp coverage was performed using the gMATS and MenDeVAR Index methods, respectively. Although the MenDeVAR Index also estimates reactivity of 4CMenB, in contrast to gMATS, this method includes NadA peptide as a predictor (Rodrigues et al., 2020). We were not able to detect the *nadA* allele using targeted PCR for some isolates (**Supplementary Table 1**); therefore, 4CMenB coverage was not assessed using the MenDeVAR Index. Although the methods use slightly different criteria for defining cross-reactive antigens (Muzzi et al., 2019; Rodrigues et al., 2020), 49 (84.5%, CI 73.1-91.6%) isolates were potentially covered by both vaccines (comprising ‘amber’ cross-reactive antigens for evaluation by the MenDeVAR Index). Seven isolates showed diverging vaccine coverage predictions by the Fhbp antigen, which mostly contributed to the potential protection of 4CMenB (**Table 6**).

**Table 6:** 4CMenB and MenB-Fhbp vaccine coverage predictions by the Fhbp antigen.

Isolate id	Sequence type	Clonal complex	Fhbp peptide variant	gMATS (4CMenB vaccine)	MenDeVAR Index (MenB-Fhbp vaccine)
3579	ST-5477	ccUA	13	Unpredictable	Covered (cross-reactive)
3906	ST-34	32	180	Unpredictable	Covered (cross-reactive)
13530	ST-16207	ccUA	19	Not covered	Covered (cross-reactive)
21577	ST-4769	cc18	37	Covered	Insufficient data
22805	ST-15982	ccUA	119	Not covered	Insufficient data
29852	ST-10261	ccUA	647	Not covered	Insufficient data
38997	ST-9774	cc18	37	Covered	Insufficient data

## 4 Discussion

During the study period (2009-2019), Lithuania, along with more populated and distant countries such as the United Kingdom and Ireland, ranked high in number of IMD cases per 10^5^ population among European Union/European Economic Area countries (Ladhani et al., 2012; Bennet et al., 2019; European Centre of Disease Prevention and Control, 2022). The incidence of IMD was significantly lower in neighboring Lithuania countries such as Poland, Latvia, and Estonia accounting for 0.36, 0.6, and 0.3 cases per 10^5^ population, respectively (European Centre of Disease Prevention and Control, 2022). In 2018-2019, the incidence of IMD decreased in Lithuania (**Table 1**), but we assume that it is more related to the temporal patterns associated with the disease (Mosavi-Jarrahi et al., 2009) than to the effect of vaccination. 4CMenB vaccination started in the middle of 2018.

Although the clinical practice of IMD management is based on culture proven-diagnosis (Diagnostic and Treatment Protocols, Ministry of the Republic of Lithuania, 2015), invasive meningococcal isolates stored at the NPHSL account for approximately 40% of all reported disease cases from 2009 to 2019, which is much less than in Poland, the Netherlands, and Finland (Bodini et al., 2020; Freudenburg-de Graaf et al., 2020; Waśko et al., 2020). Several studies report low recovery (35-43%) of *N. meningitidis* from sterile sites of the human body (Bronska et al., 2006; Heinsbroek et al., 2013; Lee et al., 2018). It needs to be clarified whether the missed isolates are due to difficulties in recovering *N. meningitidis* from the samples, neglect of mandatory sending of invasive isolates to the NPHSL, or the increasing number of PCR-confirmed IMD cases.

During the period of 2009-2019, serogroup B of invasive *N. meningitidis* was the most common serogroup (90.5% of isolates), whereas MenC accounted for 7.5% of isolates. A small number of serogroup Y and W isolates were from cases diagnosed after 2015. In Lithuania, MenY and MenW cases were found only in adults, consistent with findings from Canada (Tsang et al., 2020) and the Netherlands (Freudenburg-de Graaf et al., 2020).

MLST analysis revealed the dominance of cc32 (74.02%), which has been found globally and is widespread in the Netherlands (32%) (Freudenburg-de Graaf et al., 2020), Czech Republic (29%) (Krizova and Honskus, 2019), France (27.5%) (Hong et al., 2021), Poland (20.1%) (Waśko et al., 2020), Germany (21.6%), Norway (17.1%), and Italy (14.8%) (Vogel et al., 2013). In the past, the hypervirulent cc32 caused outbreaks in Norway, Cuba, France, the Netherlands, and the United Kingdom (Harrison et al., 2009; Racloz and Liuz, 2010). There are no data on invasive meningococcal isolates in neighboring and demographically similar countries Latvia and Estonia (https://pubmlst.org/neisseria/, accessed December 2022).

Clonal complex 41/44 was the second most dominant cc (6.4%) with strains associated with serogroups B and C, which confirms their propensity to rapid evolutionary changes in serotypes and genotypes (Whelan et al., 2015). Isolates of cc41/44 were representative of 14 different STs, with ST-2871 being the most common, although it is found in very few countries (https://pubmlst.org/neisseria/, accessed December 2022). We detected only three isolates that belong to the hyperinvasive cc11 potentially associated with high morbidity and mortality (Lucidarme et al., 2015).

Among the cc32 isolates, ST-34 was the most frequently detected ST among the invasive meningococci in Lithuania. Finetyping of PorA and FetA variable regions showed that the strain B: P1.19,15: F4-28: ST-34 (cc32) accounted for 64.1% of all analyzed meningococcal isolates. The distribution of this strain remained almost stable over the study period. According to PubMLST *Neisseria* data, this invasive strain was recovered in a small number of countries, including England (n=17 isolates, 2010-2020), Poland (n=3, 2012), France (n=2), and Spain (n=2). Thus, the hypervirulent strain B: P1.19,15: F4-28: ST-34 (cc32) has not spread to other countries but instead is well-established in Lithuania. This strain was found among all age groups, most commonly among 5-14- and 15-24-year-olds. The characteristic feature of the prolonged cc32 MenB epidemics in Cuba and Norway (1980s) and in Oregon in the United States (1990-2000s) was the predominance of a single bacterial strain (>85%) rather than a mixture of heterologous strains (Diermayer et al., 1999; Harrison et al., 2009). Keeping in mind that isolates from approximately 50% of IMD cases in Lithuania were not included in the analysis, the prevalence of the strain B: P1.19,15: F4-28: ST-34 (cc32) may be even higher than detected.

Given that vaccination against MenB is widely accessible in Lithuania, genetic methods were used to assess the potential coverage of the most recent MenB isolates by the available vaccines. The current study shows that the predicted strain coverage by 4CMenB using gMATS was 91.4% (CI 85.9-98.2%). Only a few isolates possessed 4CMenB-covered Nhba or PorA variants (**Table 4**, **Supplementary Table 1**). In Lithuanian isolates, the Nhba non-covered variant 187 was linked with cc32, whereas in Polish isolates, peptides 3 and 20 were mostly detected in cc32 (Waśko et al., 2020). A high proportion of isolates (84.5%) was predicted to be covered by a single 4CMenB vaccine antigen, Fhbp, most often by its variant 1. This peptide variant is associated with cc32 (Freudenburg-de Graaf et al., 2020; Waśko et al., 2020); therefore, it is expected that the predominant ST-34 (cc32) is also potentially covered by the two-component MenB-Fhbp vaccine due to its cross-reactivity (i.e., ‘amber’ MenDeVAR Index).

Some Lithuanian MenB isolates (9.0%) were not assigned to any clonal complex, whereas isolates of ccUA accounted for 22% in Norway, 19.4% in Germany, 9.3% in Italy (Vogel et al., 2013), 20.2% in Poland (Waśko et al., 2020), and 6.1% in Greece (Tzanakaki et al., 2021). The predicted coverage data of isolates collected from 2017 to 2019 showed that the antigen peptide variants present in ccUA strains often fall into the grey zone characterized by ‘insufficient data’ or ‘unpredictable’ categories according to the gMATS and/or MenDeVAR Index methods (Mulhall et al., 2018; Moreno et al., 2020). The Lithuanian isolates of ccUA were unevenly distributed across age groups, with the highest proportion found among children < 1-year-old (**Figure 2**). Children of this age are potential targets for vaccination; therefore, attention should be paid to obtaining and analyzing more isolates of IMD cases from this age group. Participation in the Global Meningococcal Initiative in Eastern Europe (Bai et al., 2019) and other surveillance projects would open new possibilities for cooperation with other study groups to apply MATS and meningococcal antigen surface expression (MEASURE), assays for coverage prediction of novel STs along with genomic data to update the gMATS and MenDeVAR Index databases.

One limitation of this study is that all individuals eligible for molecular typing of meningococcal isolates accounted for less than one-half of all reported IMD cases. We encourage clinicians to take blood and CSF samples for culturing if possible before initiating antibiotic therapy. Second, the number of MenB isolates of IMD cases subjected to vaccine-predicted coverage estimation was relatively small and did not allow evaluation of age-specific distributions. Third, the absence of genomic characterization of *nadA* for some MenB isolates did not permit use of the MenDeVAR Index method to predict their coverage by the 4CMenB vaccine.

In conclusion, the study provides the first comprehensive genetic characterization of invasive meningococcal isolates collected in Lithuania from 2009 to 2019. The vast majority of isolates belonged to serogroup B, and the most prevalent cc was cc32. The strain B: P1.19,15: F4-28: ST-34 (cc32) accounted for more than one-half of all collected invasive isolates over the study period. This strain is rare in neighboring and distant European countries. Application of well-recognized genetic tools revealed that 84.5% (CI 73.1-91.6%) of isolates collected in 2017-2019 were covered by both MenB vaccines. Assessment of strain coverage by MenB vaccines using genomic analysis of vaccine-related antigens is convenient and valuable, as not all countries may perform MATS assays. The continuation of active IMD surveillance in Lithuania is needed to monitor changes in circulating meningococcal strains that would allow evidence-based implementation of a national vaccination strategy.

## Data availability statement

The data presented in the study are available in the publicly accessible PubMLST Neisseria repository (https://pubmlst.org/neisseria). The names of accession numbers can be found below: 110094-110096, 110181, 110183, 110185-110188, 110190-110194, 110196-110204, 110206, and 110207.

## Ethics statement

The studies involving human participants were reviewed and approved by the Vilnius Regional Biomedical Research Ethics Committee (http://www.eurecnet.org/information/lithuania.html), approval no. 2021/9-1369-846, issued 21-09-2021. Written informed consent for participation of children was not provided by the participants’ legal guardians/next of kin because: this is a retrospective study that included all meningococcal isolates from invasive meningococcal disease received in the National Public Health Surveillance Laboratory from 2009 to 2019.

## Author contributions

AZ and MP contributed to the conception and design of the study. ES, RP, AP, DS, IK-K, and MP performed the experiments. VG performed the statistical analysis. MP, AZ, and II wrote the manuscript. MP and AP submitted data to the PubMLST *Neisseria* database. All authors contributed to the manuscript revision and approved the submitted version.
